# Asymmetry induced suppression of chaos

**DOI:** 10.1038/s41598-020-72476-8

**Published:** 2020-09-24

**Authors:** Animesh Biswas, Sudhanshu Shekhar Chaurasia, P. Parmananda, Sudeshna Sinha

**Affiliations:** 1 Department of Physics, Indian Institute of Technology, Bombay, Powai, Mumbai, 400 076 India; 2 Department of Physical Sciences, Indian Institute of Science Education and Research Mohali, Sector 81, Manauli, PO 140 306 Punjab India

**Keywords:** Applied physics, Statistical physics, thermodynamics and nonlinear dynamics

## Abstract

We explore the dynamics of a group of unconnected chaotic relaxation oscillators realized by mercury beating heart systems, coupled to a markedly different common external chaotic system realized by an electronic circuit. Counter-intuitively, we find that this single dissimilar chaotic oscillator manages to effectively steer the group of oscillators on to steady states, when the coupling is sufficiently strong. We further verify this unusual observation in numerical simulations of model relaxation oscillator systems mimicking this interaction through coupled differential equations. Interestingly, the ensemble of oscillators is suppressed most efficiently when coupled to a completely dissimilar chaotic external system, rather than to a regular external system or an external system identical to those of the group. So this experimentally demonstrable controllability of groups of oscillators via a distinct external system indicates a potent control strategy. It also illustrates the general principle that symmetry in the emergent dynamics may arise from asymmetry in the constituent systems, suggesting that diversity or heterogeneity may have a crucial role in aiding regularity in interactive systems.

## Introduction

A fixed point of a dynamical system, also commonly known as an equilibrium or steady state, is a state where all variables are constant in time. Steady states are often the desired target of complex systems comprised of mechanical^1^, optical^2,3^, thermo-optical^4^, electrical, chemical^5,6^ and biological oscillators^7^. So, the unearthing mechanism that can guide complex dynamics to stable fixed states is of considerable relevance, both for understanding the diverse ways in which intrinsic complexity can be reigned, as well as for applications that rely on stabilization of steady states^8–21^. Conversely, the enhancement of chaos also has important practical engineering applications^22^, and in that context too it is valuable to find underlying mechanisms that may have lead to the undesirable steady states.

Here we consider the dynamics of an ensemble of uncoupled oscillators, coupled only to a common external oscillator. That is, the oscillators in the group have no direct coupling amongst themselves, and the interaction is wholly mediated via the external system^23,24^. So this common oscillator can be considered analogous to a pacemaker or pacesetter, with feedback, driving the group of oscillators. We examine the scenario where the external oscillator is intrinsically chaotic and has qualitatively different dynamics arising from a class of systems quite distinct from those comprising the oscillator ensemble.

In the sections below we first present experimental results demonstrating the taming of chaos by coupling to an external markedly different chaotic system. Specifically our oscillator group is comprised of chaotic mercury beating heart electrochemical oscillators, and the external system is a chaotic electronic circuit. We further verify our central experimental result through extensive numerical simulations on a model system of coupled differential equations suggestive of the experimental set-up. We conclude with discussions on the general scope and implications of these results.

## Experimental demonstration of asymmetry induced chaos suppression

**Figure 1:**
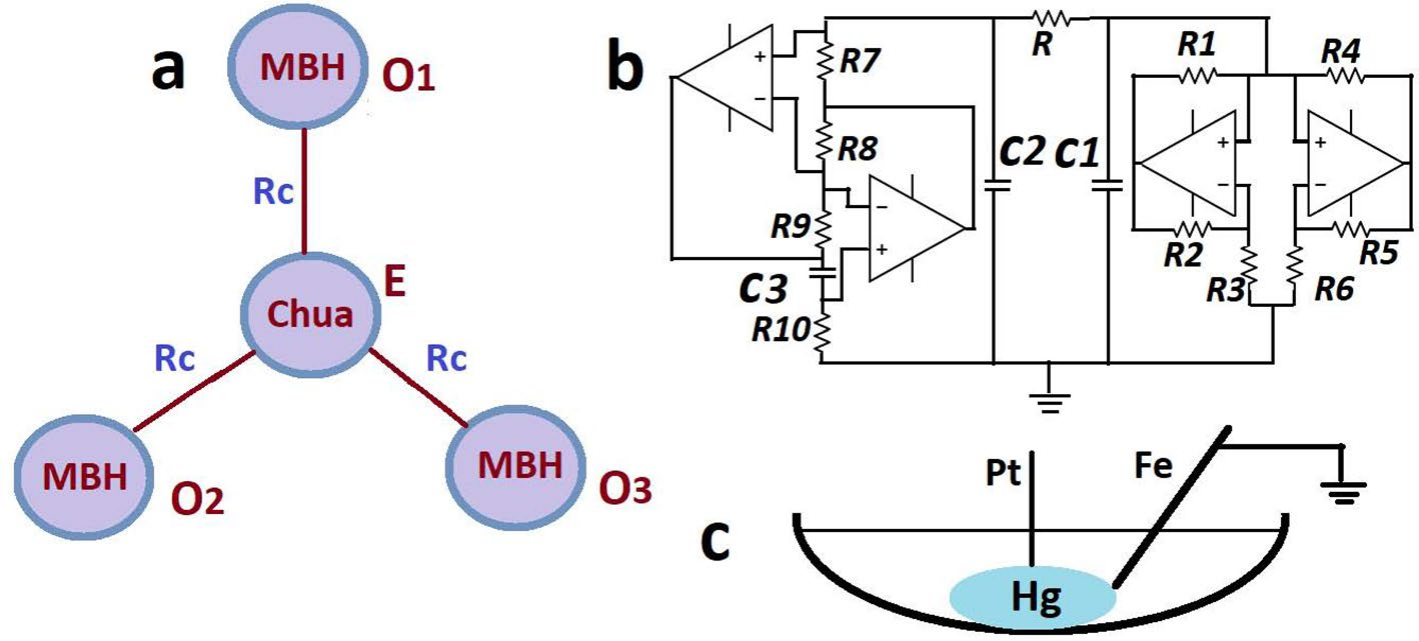
The experimental setup. (**a**) Schematic diagram of three surrounding MBH oscillators ($O_1, O_2, O_3$) are connected to an external Chua oscillator (E). (**b**) The circuit diagram of an inductor-free Chua oscillator. The inductor and Chua diode are implemented by using op-amps, resistors, and capacitors. (**c**) A schematic diagram of an MBH oscillator. The coupling between the MBH oscillators and Chua are made via resistance ($R_c$), and the inverse of resistance is the measure of coupling strength.

We consider three mercury beating heart (MBH) oscillators ($O_1, O_2, O_3$) connected to an external Chua oscillator (E), as shown in the schematic diagram in Fig. 1a. Figure 1b shows the circuit diagram of an inductor-free Chua oscillator and Fig. 1c shows the schematic of an MBH oscillator. The group of MBH oscillators are bidirectionally coupled to the external oscillator via resistance ($R_c$), where the inverse of the $R_c$ is the measure of coupling strength. The coupling between the Chua and MBH oscillators and the circuit of Chua oscillator are implemented on the breadboard using electronic components such as resistors, capacitors, and op-amps (741-IC).

In an MBH system, a concave watch glass of radius of curvature of 7.56 cm is used to contain the mercury drop (Hg) and an aqueous electrolytic solution. One millilitre volume of mercury is placed on the watch glass and immersed under an electrolytic solution. Here, the electrolytic solution is a mixture of 10 ml of 6 M H_2_SO_4_ (Merck, Emparta ACS, 98.0%) and 15 ml of 0.2 M Ce(SO_4_)_2_ (Sigma Aldrich). Before each experiment, we cleaned the mercury following the protocol described in the Ref.^25^. A pointed iron (Fe) nail (2 mm of diameter, purity $\ge \, 99.0\%$, Alfa Aesar) is placed at an appropriate position near the Hg drop to start the oscillations on the drop. The iron nail is polished with a sandpaper No. 1000 before the experiments. Moreover, the iron nail is fixed near the upper edge of the Hg drop to get the sustained irregular aperiodic oscillations. A Platinum (Pt) wire (Sigma-Aldrich, 0.5 mm of diameter, 99.99% purity) is submerged in the center of the drop to provide electrical contact. Three such MBH oscillators exhibiting chaotic oscillatons are used in our experiments. All the MBH oscillators have a common ground via iron nails. The signals (electrode potentials) are recorded between the Pt wires and Fe nails using the data acquisition control card (DAQ measurement computing USB-1616HS-4, 1000 Hz) with a computer interface. The details of the underlying nonlinear dynamics and chemistry of the regular and chaotic MBH oscillator can be found in the relevant literature^26–33^. Specifically, we consider the MBH oscillators in the parameter range that has been shown to yield chaotic dynamics^32^.

In Fig. 1b, the schematic diagram of the Chua oscillator shows the circuit implementation of the simulated inductor and Chua diode by using op-amps (741-IC), resistors ($$R_1-R_{10})$$, and capacitors ($$C_1, C_2,$$ and $$C_3$$). The parameter values for the circuit are taken from the paper by Torres et al.^34^. Few parameter values are different such as $$C_3 = 3.3 \, \mu \hbox {F}$$, $$C_1 = 3.3 \, \mu \hbox {F}$$, $$C_2 = 47 \, \mu \hbox {F}$$, and $$R = 1.73 \, \hbox {k} \Omega $$ in our experiments. For other components, see Ref.^34^. The parameter *R* controls the periodic/chaotic dynamics of the oscillator. The signals of the oscillator are the corresponding voltages $$V_1$$ and $$V_2$$ across the capacitors $$C_1$$ and $$C_2$$. According to the circuit components, the variable $$V_1$$ shows double scroll chaotic dynamics, and $$V_2$$ shows single scroll chaotic dynamics. In our experiments, we couple the variable $$V_2$$ to the group of chaotic MBH oscillators. The Chua oscillator is also grounded at the common ground of the MBH oscillators.

**Figure 2:**
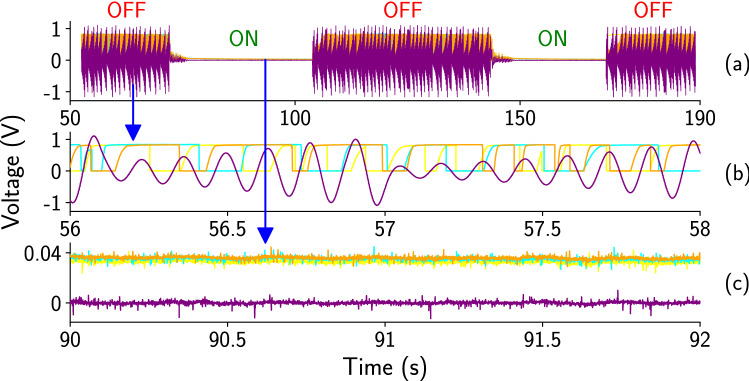
The redox time series of three chaotic MBH oscillators (Cyan, orange, and goldenrod) and the single scroll Chua oscillator (purple color) are shown. (**a**) Superimposed time series of all the four oscillators for the section of coupling ‘OFF’–‘ON’–‘OFF–‘ON’–‘OFF’ state at fixed coupling value ($R_c = 40 \, \Omega$). (**b**) Uncorrelated dynamics of the oscillators while the coupling is ‘OFF’. (**c**) Fixed point dynamics of all the oscillators at coupling ‘ON’ state. The fixed point values of the MBH oscillators are close to 0.04, and for Chua, the fixed point value is 0.

We now present the emergent dynamics of three surrounding chaotic MBH oscillators bi-directionally coupled to a common external chaotic Chua oscillator. The single-scroll chaotic signal $V_2$ of the Chua oscillator is coupled to the MBH oscillators, and the suppression of oscillations of the entire system is explored as a function of coupling strength ($\frac{1}{R_c}$). Figure 2a shows a superimposed time series of all the four oscillators (Chua and MBH oscillators) from an experimental trial. The coupling is turned ‘OFF’ and ‘ON’ two times systematically at a fixed coupling strength ($R_c = 40 \, \Omega$). Figure 2b shows the dynamics for MBH oscillators and the chaotic Chua oscillator when the coupling is ‘OFF’. Figure 2c shows the fixed point dynamics of all four oscillators when the coupling is ‘ON’.

**Figure 3:**
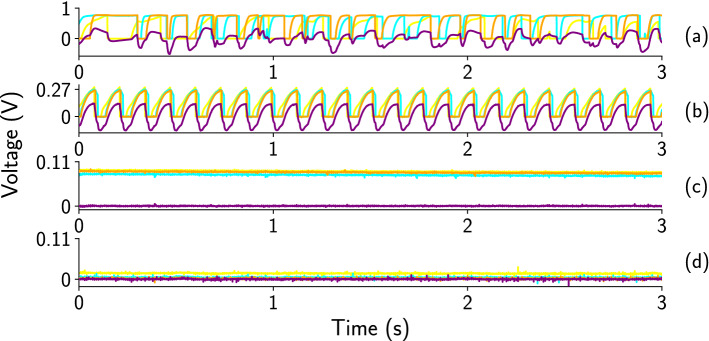
The dynamics of the three surrounding MBH oscillators and the central Chua oscillator are shown as a function of coupling strength ($\frac{1}{R_c}$). (**a**) For weak coupling ($R_c = 270 \, \Omega$), the dynamics of all the coupled oscillators are uncorrelated and irregular. (**b**) At moderate coupling strength ($R_c = 200 \, \Omega )$, oscillators show synchronized period-1 dynamics. (**c**) Oscillations of all the coupled oscillators are suppressed, and they show fixed point dynamics at coupling resistance value $R_c = 100 \, \Omega$. The fixed point values for the MBH oscillators are close to 0.1, and for the Chua, the value is close to 0. (**d**) At $R_c = 2 \, \Omega$, the dynamics are still fixed point, but the fixed point values of MBH oscillators now close to 0.

The dynamics of the coupled Chua and the MBH oscillators show the transitions from uncorrelated irregular oscillations to synchronized regular oscillations and fixed-point dynamics as the coupling strength ($\frac{1}{R_c}$) increases. For weak coupling ($R_c = 270 \, \Omega$), the suppression of oscillations does not occur, and the dynamics of the oscillators are uncorrelated (Fig. 3a). As the coupling strength increases, we find period-1 oscillations (Fig. 3b) and fixed-point dynamics (Fig. 3c–d) for all the coupled oscillators (MBH and Chua oscillators). For moderate coupling strength ($R_c = 200 \, \Omega$), oscillators show synchronized period-1 oscillations (Fig. 3b). Fixed point dynamics of the oscillators are observed within a range of coupling strength ($R_c = 2 \, \Omega$ to $100 \, \Omega$). The fixed point values for the MBH oscillators are different for different coupling strengths, reminiscent of *oscillation death*. For $R_c = 100 \, \Omega$, the fixed point values for the MBH oscillators are close to 0.1 (Fig. 3c). For strong coupling strength ($R_c = 2 \, \Omega$), the fixed point values for MBH oscillators are close to 0 (Fig. 3d). The fixed point value for the single scroll Chua is 0 for both $R_c = 100 \, \Omega$ and $2 \,\Omega$.

**Figure 4:**
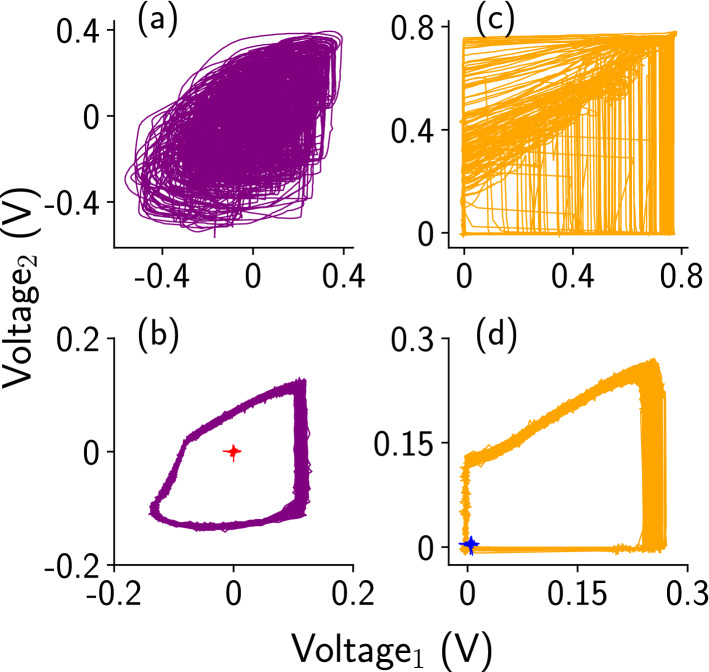
Phase space plots of the uncontrolled and controlled dynamics of the Chua and one representative MBH oscillator. (**a**) For weak coupling, uncontrolled dynamics of the Chua oscillator ($R_c = 270 \, \Omega$). (**b**) Controlled period-1 dynamics (purple color) and fixed point dynamics (red mark) of Chua oscillator for $R_c = 200 \, \Omega$ and $R_c = 2 \, \Omega$, respectively. (**c**) Uncontrolled dynamics of one representative MBH oscillator at weak coupling ($R_c = 270 \, \Omega$). (**d**) Controlled period-1 (orange color) and fixed point (blue mark) dynamics of an MBH oscillator at $R_c = 200 \, \Omega$ and $R_c = 2 \, \Omega$, respectively.

Finally, the phase space plots of the uncontrolled and controlled dynamics of the Chua and one representative surrounding MBH oscillator are shown in Fig. 4 for different coupling strengths. The data plotted in the phase plots correspond to the time series data in the Fig. 3. Figure 4a shows the uncontrolled dynamics of the Chua oscillator at weak coupling ($R_c = 270\, \Omega$). The controlled period-1 (purple color) and fixed point (red dot) dynamics of Chua are shown in Fig. 4b for $R_c=200 \, \Omega$, and $R_c=2 \, \Omega$) respectively. For the oscillator group, the phase space plot is shown for one representative MBH oscillator in Fig. 4c,d. Figure 4c shows the uncontrolled chaotic dynamics of the MBH oscillator and Fig. 4d shows the controlled period-1 (orange color) and fixed point (blue dot) dynamics for $R_c=200 \, \Omega$, and $R_c=2 \, \Omega$ respectively.

## Numerical simulations

We now explore the dynamics of a group of relaxation oscillators coupled to a single external chaotic Chua system. While serving as an illustrative toy model mimicking the experimental setup above, this model system also allows us to systematically vary the size of the group of oscillators and coupling strengths over a large range, thus enabling us to explore a broader scenario. Specifically we examine the dynamics of *N* oscillators in the group, labelled by index $i= 1, \dots N$, given by the FitzHugh-Nagumo relaxation oscillator equations,

1 $$\begin{aligned} \frac{d x_i}{dt}= & {} x_i - \frac{x_i^3}{3} - y_i + I + \ C \{ y_{ext}-x_i \} \nonumber \\ \tau \frac{d y_i}{dt}= & {} x_i - b \ y_i + a \end{aligned}$$

where ($x_i$, $y_i$) are the state variables of the *i*th oscillator of the group, with $x_i$ mimicking the voltage and $y_i$ representing the slower recovery current. As in the experiment, the dynamical variable $y_{ext}$ of the external oscillator is the only state variable that couples the external system to the oscillator group, with parameter *C* determining the strength of coupling. Note again that there is no mutual coupling among the relaxation oscillators, which couple bidirectionally only to the common external system. Further note that in the experiment the coupling between oscillators is proportional to the potential difference between the oscillators, and so the form of the coupling is well modelled as being diffusive.

The external chaotic system, which is distinct from the group, is considered to be the paradigmatic chaotic Chua system, given by the dynamical equations

2 $$\begin{aligned} \frac{d x_{ext}}{dt}= & {} \alpha \{ y_{ext}-x_{ext}-g(x_{ext}) \} \nonumber \\ \frac{d y_{ext}}{dt}= & {} \{ x_{ext}-y_{ext}+z_{ext} \} + \ C \sum _{j=1}^N \{ x_j-y_{ext} \} \nonumber \\ \frac{d z_{ext}}{dt}= & {} - \beta y_{ext} \end{aligned}$$

where $g(x) = m_1 x + 0.5 (m_0-m_1) (|(x+1)|- |(x-1)|)$. A wide set of parameters that yielded chaotic attractors were examined, and we present results from a representative system with parameters $\alpha =15.6$, $\beta =28$, $m_1=-5.0/7.0$ and $m_0=-8.0/7.0$ in Eq. (2).

**Figure 5:**
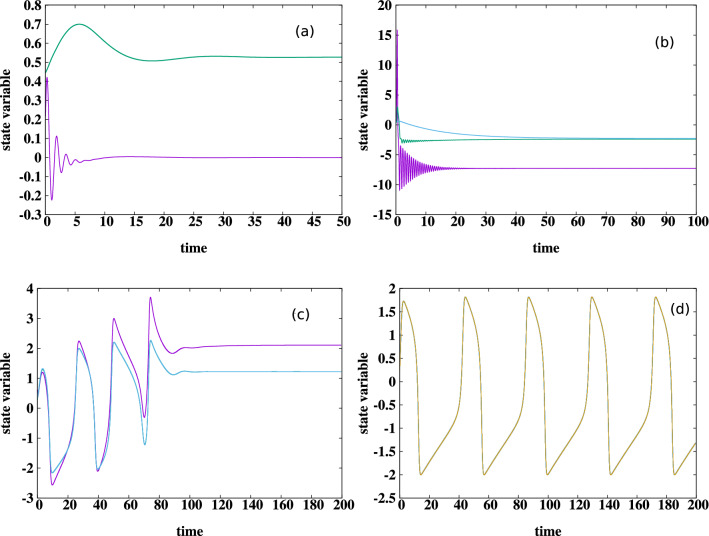
Time evolution of one representative relaxation oscillator in the group described by Eq. (1), with parameters $a=0.8$, $b=0.7$, $I=0.5$ and $\tau = 12.5$, when coupled to the following external systems: (**a**) chaotic Chua system ($C=1$, with the external system coupled to the oscillator group through variable $y_{ext}$); (**b**) chaotic double-scroll Lorenz system ($C=0.5$, with the external system coupled to the oscillator group through variable $x_{ext}$); (**c**) chaotic single-scroll Rössler system ($C=2$, with the external system coupled to the oscillator group through variable $x_{ext}$); (**d**) identical relaxation oscillator ($C=5$, with the external system coupled to the oscillator group through variable $x_{ext}$). Here the *x* variable of all the oscillators and $x_{ext}$ of the external system are displayed. Clearly the first three cases, where the external system is chaotic and dissimilar go to fixed points, while coupling to an identical external system yields synchronized oscillations but no steady states.

**Figure 6:**
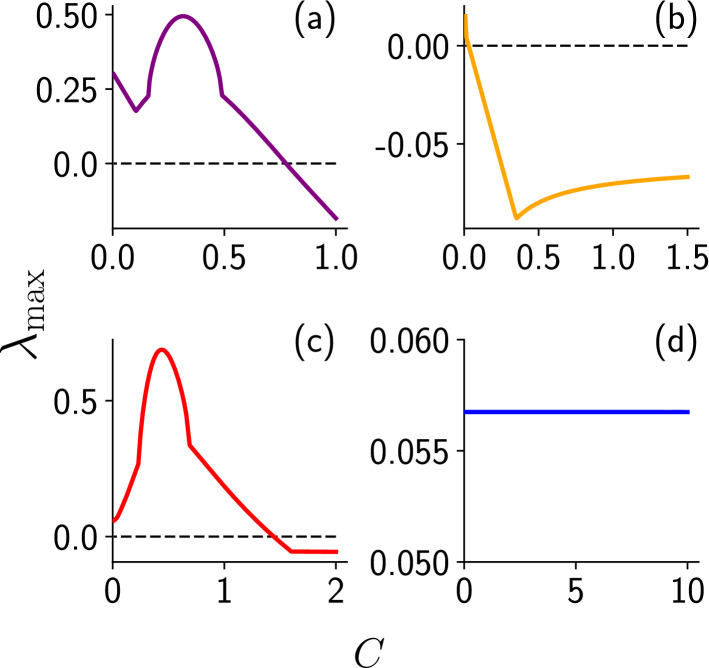
Maximum real part $\lambda _{max}$ of the eigenvalues of the Jacobian at the fixed points, as a function of coupling strength *C*, for the case of a group of three FtizHugh-Nagumo relaxation oscillators (cf. Eq. (1)) coupled to the following external systems: (**a**) chaotic Chua system, (**b**) chaotic Lorenz system, (**c**) chaotic Rössler system, and (**d**) an identical FitzHugh-Nagumo relaxation oscillator. These four cases correspond to the external systems considered in the time series displayed in Fig. 5a–d. It is clear that after a threshold coupling strength the eigenvalues are negative for cases (**a**–**c**), signalling the emergence of a stable steady state for sufficiently strong coupling when the external system is dissimilar. However in case (**d**), where the external system is identical, notice that $\lambda _{max}$ is positive and remains constant under increasing coupling strength (which is a consequence of the vanishingly small contribution of the coupling term when the oscillators are close to synchrony), and this indicates that no stable fixed points emerge in that case.

When the coupling is weak, the oscillators in the group retain their relaxation oscillations, and the external system maintains its chaotic behaviour. However remarkably, as displayed in representative examples in Fig. 5a, steady states emerge when the the ensemble of relaxation oscillators interact strongly with the external chaotic system. As a reference note that the same relaxation oscillators when coupled to an external relaxation oscillator of the same type do not yield steady states, even at very high coupling strengths (see Fig. 5d). These numerical trends are also corroborated by linear stability analysis obtained through the analysis of the eigenvalue spectrum of the Jacobian (see Fig. 6). We varied the number of oscillators *N* in the group from one to twenty, and found similar qualitative behaviour, with the specific window of coupling strength in which steady states emerge depending on *N*.

In order to explore a broader set of systems we simulated the dynamics of this group of oscillators coupled to an external single-scroll chaotic Rössler system and an external double-scroll chaotic Lorenz system, with the external system coupled to the oscillator group either through $x_{ext}$ or $y_{ext}$ of the external system (see Fig. 5 for some illustrative results). We incorporated varying time-scales of the intrinsic dynamics of the oscillators and the external system as well. Under variation of coupling strengths and the specific nature of the dynamics (such as the number of attractor scrolls) of the external system and oscillator group, we found a wide range of dynamical patterns arise. These patterns include chimera states^35–41^, bursty or irregular low-amplitude oscillations, and distorted attractors with the external system cloning the oscillator group dynamics or vice-versa. However, the broad observation that holds across systems is that an *ensemble of uncoupled oscillators, strongly coupled to a significantly dissimilar external system, induces oscillation suppression in both the oscillator group as well as the external system*. This occurs even when the external system and the oscillator group is chaotic. In fact counter-intuitively, an ensemble of oscillators are tamed more effectively by an external dissimilar chaotic system than an external regular system.

## Conclusions

In summary, we have investigated through laboratory experiments, as well as numerical simulations, the behaviour of an ensemble of uncoupled oscillators, with varying intrinsic dynamics, coupled diffusively to an external oscillator. The common external system may be similar or dissimilar to the group. We explored all possible scenarios, with the intrinsic dynamics of the external oscillator ranging from regular to chaotic. Counter-intuitively, we find that an external oscillator manages to successfully steer a group of oscillators on to steady states at sufficiently high coupling strengths when it is *distinct* from the group, rather than identical. That is, the oscillator group coupled to an external oscillator synchronizes for strong coupling, regardless of whether the external one is identical or dissimilar, regular or chaotic. However surprisingly, fixed states emerge *only* if the external oscillator is significantly dissimilar. These results then indicate the easy controllability of oscillators by coupling to an external dissimilar chaotic system, thereby offering a new potent control strategy. Since this phenomenon was observed in a generic class of systems it holds promise of having wide-ranging validity. However, we must add the caveat that its full scope and extent is as yet undetermined. So an open question here is the generality of these results under varying dynamics and coupling classes, and this warrants future work across different systems, both theoretical and experimental^42^.

Lastly, we would like to discuss these findings from a conceptual point of view. Our observations here demonstrate specific examples of the interesting general principle of *asymmetry inducing symmetry* in coupled dynamical systems^43^. That is, a coupled system constituted of exact replicas often yield irregular spatiotemporal patterns, while markedly different constituents can yield very regular and symmetric spatiotemporal patterns such as steady states or synchronized low amplitude regular oscillations. This dynamical behvaiour can also be interpreted as an *anti-chimera* state. While chimera states, which have commanded widespread research attention in recent years^35–41^, signal the emergence of asymmetric spatial patterns in a system comprised of identical dynamical elements, here we have the emergence of symmetric spatiotemporal patterns through coupling to a system that is significantly different. This further suggests that diversity or heterogeneity may have a crucial role in aiding regularity in interactive systems.
